# CircRNA-Based Cervical Cancer Prognosis Model, Immunological Validation and Drug Prediction

**DOI:** 10.3390/curroncol29110633

**Published:** 2022-10-25

**Authors:** Xu Guo, Sui Chen, Sihan Wang, Hao Zhang, Fanxing Yin, Panpan Guo, Xiaoxu Zhang, Xuesong Liu, Yanshuo Han

**Affiliations:** 1 School of Life and Pharmaceutical Sciences, Dalian University of Technology, Dalian 116024, China; 2 School of Health Professions, Yingkou Vocational and Technical College, Yingkou 115099, China

**Keywords:** cervical cancer, competing endogenous RNA, circular RNA, microRNA, immune infiltration, cancer prognosis

## Abstract

Background: Cervical cancer (CC) is a common cancer in female, which is associated with problems like poor prognosis. Circular RNA (circRNA) is a kind of competing endogenous RNA (ceRNA) that has an important role in regulating microRNA (miRNA) in many cancers. The regulatory mechanisms of CC immune microenvironment and the transcriptome level remain to be fully explored. Methods: In this study, we constructed the ceRNA network through the interaction data and expression matrix of circRNA, miRNA and mRNA. Meanwhile, based on the gene expression matrix, CIBERSORT algorithm was used to reveal contents of tumor-infiltrating immune cells (TIICs). Then, we screened prognostic markers based on ceRNA network and immune infiltration and constructed two nomograms. In order to find immunological differences between the high- and low-risk CC samples, we examined multiple immune checkpoints and predicted the effect of PD-L1 ICI immunotherapy. In addition, the sensitive therapeutics for high-risk patients were screened, and the potential agents with anti-CC activity were predicted by Connective Map (CMap). Results: We mapped a ceRNA network including 5 circRNAs, 17 miRNAs and 129 mRNAs. From the mRNA nodes of the network six genes and two kind of cells were identified as prognostic makers for CC. Among them, there was a significant positive correlation between CD8+ T cells and *SNX10* gene. The results of TIDE and single sample GSEA (ssGSEA) showed that T cells CD8 do play a key role in inhibiting tumor progression. Further, our study screened 24 drugs that were more sensitive to high-risk CC patients and several potential therapeutic agents for reference. Conclusions: Our study identified several circRNA-miRNA-mRNA regulatory axes and six prognostic genes based on the ceRNA network. In addition, through TIIC, survival analysis and a series of immunological analyses, T cells were proved to be good prognostic markers, besides play an important role in the immune process. Finally, we screened 24 potentially more effective drugs and multiple potential drug compounds for high- and low-risk patients.

## 1. Introduction

Cervical cancer (CC) is the fourth most common human cancer at present. According to statistics, there were 570,000 CC patients in the world in 2018, including 311,000 dying [1]. CC has become the second major cancer in women, only second to breast cancer (BC), and it mainly occurs in women aged 20–39 years old. Over the past few years, early screening, surgical treatment, radiochemotherapy, and targeted therapy have been proved to effectively reduce CC morbidity and mortality, but the clinical outcome and long-term survival of relapsed or advanced CC patients remain unsatisfactory, besides, there is still no effective treatment for advanced CC patients. Further, although there are many screening and early detection methods, many patients are diagnosed at the middle and advanced stages, accompanied by tumor infiltration or invasion, leading to the problems of low survival rate and poor prognosis. Consequently, it is of great clinical significance and application value to identify the diagnostic and prognostic biomarkers for CC and construct the reliable prediction model to predict the survival rate of individual CC patients.

Circular RNA (circRNA) is a kind of special non-coding RNA (ncRNA) derived from the gene intron or exon region. Due to the closed-loop structure, circRNA exhibits neither 5′-3′ polarity nor polyA tail. Consequently, compared with linear RNA, circRNA has a more stable structure and is not easily digested by RNA exonuclease or RNase R. As pointed out by the competing endogenous RNA (ceRNA) mechanism hypothesis, some ncRNAs, like circRNAs, also contain the miRNA response element, therefore, they can compete against mRNA for binding to miRNA, thus indirectly regulating mRNA expression and forming the complex post-transcriptional regulatory network [2]. To explore the potential function and regulatory mechanism of circRNA in CC, this work constructed the circRNAs-related ceRNA regulatory network. Based on the ceRNAs, we also built the prognosis risk score model to predict the overall survival (OS) of CC patients.

Existing studies have suggested that both tumor cells and immune cells participate in tumor genesis and development, and proved that immune cell types and proportions are related to survival prognosis. As confirmed in previous reports, circRNAs participate in the regulation of tumor-infiltrating immune cells (TIICs) via the ceRNA mechanism [3]. Moreover, with the development of cell molecular biology and immunology, immune therapy has become a novel treatment direction for CC. For instance, immune checkpoint (ICP) proteins CTL4 (cytotoxic T lymphocyte 4) and PD-L1 (programmed cell death 1 ligand 1) can promote CC genesis and development, while the immune checkpoint inhibitors [ICIs, including anti-PD-1 (programmed death-1)/anti-CTLA-4 (cytotoxic T lymphocyte associated antigen-4)] have been verified to exert important roles in CC treatment [4]. Consequently, this work evaluated the TIIC levels, explored the relations of ceRNA network-based risk score and TIIC levels, and carried out immune-related analyses like ICP detection and tumor mutation analysis based on the risk score. Moreover, this work also screened sensitive agents for high-risk CC patients from the known chemotherapy and targeted therapy agents, and predicted the potential agents for the immune therapy of CC by Connective Map (Cmap).

**Figure 1:**
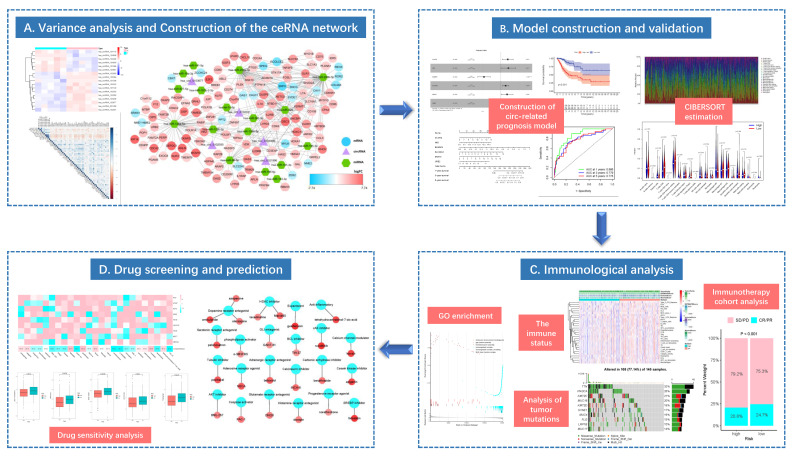
The flow diagram of this study (* *p* < 0.05; ** *p* < 0.01; *** *p* < 0.001).

This study integrated the RNA expression data and clinical information from The Cancer Genome Atlas (TCGA), Gene Expression Omnibus (GEO) and Genotype-Tissue Expression (GTEx) databases to construct the ceRNA network related to CC occurrence and development, and to explore the potential molecular mechanism. After using the “Cell type identification based on estimating relative subsets of RNA transcripts” (CIBERSORT) algorithm to evaluate the difference in immune cell composition, TIICs were combined with ceRNA network to construct a multivariate model, so as to explore the relations of prognostic genes in the model and immune cells. Additionally, for high- and low-risk populations, the immune correlation analysis was conducted using the gene set enrichment analysis (GSEA) and tumor immune dysfunction and exclusion (TIDE) algorithms. The effect of risk score model on the immune response was explored by mutation analysis and immune cohort analysis. Sensitive agents for CC patients were screened from known agents, and the potential targeted drugs with anti-CC activity were predicted by CMap. The overall flowchart of this work is presented in Figure 1.

## 2. Materials and Methods

### 2.1. Data Collection and Processing

The mRNA data of CC patients were downloaded from TCGA and GTEx databases based on the Xena database platform (https://xena.ucsc.edu/, accessed on 3 October 2021) from the University of California Santa Cruz (UCSC). Altogether 304 cancer samples (all from TCGA database) and 13 normal samples (including 10 from GTEx database and 3 from TCGA database) were downloaded. The miRNA data of CC patients were obtained from TCGA database (https://portal.gdc.cancer.gov, accessed on 3 October 2021) including 3 normal samples and 301 tumor samples which are obtained from the same set of samples as mRNA. Meanwhile, the clinical data of CC samples (*n* = 304) were downloaded from TCGA database, including age, clinical stage, tumor grade, survival time and survival status (https://portal.gdc.cancer.gov, accessed on 3 October 2021). Additionally, the circRNA data of CC patients were obtained from GEO database, which included 10 samples (5 tumor samples and 5 normal samples) and 3008 circRNA expression matrices (GSE102686). The mRNA expression data from two databases were integrated and normalized, and the names of microarray probes were transformed into gene symbols for subsequent experiments. Similarly, the expression data of circRNA and miRNA were normalized. All normalization was performed by “limma” package of R.

### 2.2. Differential Expression Analysis

The differentially expressed circRNAs (DEcircRNAs), differentially expressed miRNAs (DEmiRNAs) and differentially expressed mRNAs (DEmRNAs) between CC tissues and normal samples were screened using the limma package of Bioconductor [5] upon the thresholds of |log2(fold change, FC)| > 2 and false discovery rate (FDR) < 0.05. The differential analysis results of diverse RNAs were prepared into volcano plots and heat maps using the R package “ggpolt2” and “pheatmap”.

### 2.3. Construction of the ceRNA Network and Survival Analysis

Based on the differential analysis results, the miRNAs-mRNAs relation pairs were predicted using the StarBase v2.0 database [6]. Thereafter, the target miRNAs of DEcircRNAs were predicted based on the circRNAs-miRNAs relation data in the circBank database, and the intersected target DEmiRNAs were obtained from the above-mentioned miRNAs-mRNAs relation pairs [7]. Using *p* < 0.05 as the screening criterion, the predicted relation pairs were screened. To verify the strong correlation among mRNAs-miRNAs pairs obtained from the relation database, the expression levels of mRNAs and miRNAs from the above-mentioned relation pairs in CC samples were subject to correlation analysis. As miRNA bound to target mRNA to suppress its translation or degradation, theoretically speaking, the up-regulation of miRNA expression led to the down-regulation of corresponding mRNA accordingly. Consequently, the correlation (cor) was set at cor <−0.3, and the final miRNAs-mRNAs and miRNAs-circRNAs relation pairs were obtained to construct the ceRNA network. Later, the circRNA-miRNA-mRNA network was visualized using Cytoscape v.3.7. [8]. Additionally, this work also conducted Kaplan-Meier (K-M) survival curve analysis to evaluate the impact of genes in the circRNAs-related ceRNA network on the prognosis of CC patients.

### 2.4. Protein-Protein Interaction (PPI) Network

To investigate the interactions of products encoded by the network node genes, the PPI network was constructed by searching the STRING (the interaction genes/proteins search tool) database (http://www.string-db.org, accessed on 21 October 2021) and MCODE (Molecular Complex Detection) plug-in of Cytoscape software [9].

### 2.5. Functional Enrichment Analysis

The differentially expressed transcriptome genes, network node genes and hub genes were subject to Gene Ontology (GO) [10] functional annotation and Kyoto Encyclopedia of Genes and Genomes (KEGG) [11] pathway enrichment analyses using the “clusterProfiler” package, so as to evaluate the biological processes (BPs), molecular functions (MFs) and cellular components (CCs), as well as the signaling pathways enriched by these genes. The screening criterion was adjusted *p*-value < 0.05.

### 2.6. The Gene Cox Proportional Hazard Regression Model

Firstly, univariate Cox regression analysis was conducted to screen significantly differential genes, which were later incorporated into the initial Cox model. Subsequently, to prevent model overfitting, the least absolute shrinkage and selection operator (LASSO) regression model was adopted to select genes with the lowest cross-validation point to construct the gene model. Finally, the OS nomogram of CC patients was generated from the multivariate Cox proportional hazard regression model. To evaluate the discrimination and accuracy of the nomogram, the receiver operating characteristic (ROC) and calibration curves were plotted. In addition, the risk score of each patient was calculated, and then the patients were divided into high-risk and low-risk groups according to the median risk score for K-M survival curve analysis.

### 2.7. Evaluation of TIIC Levels and Survival Analysis

To explore the impact of risk score model on TIIC levels and the relations of key genes in the ceRNA network with immune cells, the CIBERSORT algorithm [7], which characterizes complex tissue and cell components based on gene expression profiles, was utilized to evaluate the proportions of 22 immune cells in 304 tumor tissues and 13 normal tissues [12]. Only samples of *p*-value < 0.05 were enrolled for further analysis. Meanwhile, TIICs of different stages were also analyzed for their infiltration levels. The relations between different TIIC levels and the OS of CC patients were evaluated by the K-M survival curve.

### 2.8. Immune Cell Cox Proportional Hazard Regression Model

Similar to the construction of gene prognostic model, immune cells with significant differences in univariate Cox regression analysis were integrated into the initial Cox model. Thereafter, cells with the lowest cross-validation point were selected by the Lasso regression model to construct the new model, so as to prevent model overfitting. Then, another nomogram was built based on the multivariate Cox regression model, and the nomogram discrimination and accuracy were illustrated by ROC and calibration curves. According to the median risk score, patients were divided into high- and low-risk groups for K-M survival analysis. At last, the relations of key genes with immune cells were explored by co-expression analysis.

### 2.9. Gene Set Enrichment Analysis (GSEA)

Both the high- and low-risk groups were conducted gene differential analysis at first, and 50 genes with the greatest and another 50 with the lowest logFC values were screened. In other words, 50 most significantly up-regulated genes from the high-risk group, and another 50 most significantly down-regulated genes from the low-risk group were selected for GSEA (http://software.broadinstitute.org/gsea/index.jsp, accessed on 3 October 2021). The GSEA results were obtained using the R packages “GSVA” and “GSEABase” [13].

### 2.10. Evaluation of Immune Status between High- and Low-Risk Groups

The 29 immune-related gene signatures were checked, and the immune activities in high- and low-risk groups were quantitatively evaluated by single sample gene set enrichment analysis (ssGSEA) [14]. At the same time, the ESTIMATE algorithm [15] was adopted to evaluate the corresponding tumor purity, and the differential expression of human leukocyte antigen (HLA) [16] gene family between high- and low-risk groups was further analyzed. Moreover, the TIDE algorithm was employed to predict the response to immune checkpoint blockade (ICB) immunotherapy between the high- and low-risk groups [17].

### 2.11. Tumor Mutation Analysis

Tumor mutation burden (TME) is the sum of mutations per megabase in tumor tissue. Mutation data of mutation annotation format (MAF) of CC patients were downloaded from TCGA database, then the tumor variation data were analyzed and visualized using the R package “maftools” [18]. The TMB score of each CC patient was calculated by the formula below, TMB = (total mutation number/total base number covered) × 10^6^. The top 10 genes with the highest TMB values and mutation numbers were compared between high- and low-risk groups, and displayed in the boxplots and two waterfall plots. Tumor patients with higher TMB value had better immune response [19].

### 2.12. Immunotherapy Cohort Analysis

The “IMvigor210CoreBiologies” [20] package of R language was downloaded and installed to obtain the immunotherapy cohort data of bladder urothelial carcinoma (BLCA). For each sample in the IMvigor210 cohort, the risk score was calculated according to the Cox regression coefficient during our model construction process. Thereafter, the samples were grouped based on the median risk score into high- and low-risk groups for survival differential analysis. Besides, the difference in anti-PD-L1 immunotherapeutic effect between two groups was also analyzed [21].

### 2.13. Drug Sensitivity Analysis

To search for the more sensitive therapeutics for high-risk patients, the “pRRophetic” package was utilized to convert the gene expression matrix into the half maximal inhibitory concentration (IC50) data matrix of corresponding antitumor agents [22]. Afterwards, the difference in IC50 values between high- and low-risk groups was compared by Wilcoxon test to evaluate the sensitivity to therapeutics. The results are displayed in boxplots.

### 2.14. CMap Analysis

CMap (https://clue.io, accessed on 12 January 2022) is a biological application database associating gene expression with diseases, which helps the researchers to rapidly screen small-molecular compounds or agents highly correlated with the disease based on the gene expression data, and is promising to discover novel therapeutics. In this study, gene differential expression analyses of high- and low-risk groups were performed, and differentially expressed genes (DEGs) of |logFC| > 1 were subject to correlation analysis with patient survival. Altogether 19 genes of *p*-value < 0.05 were obtained. Subsequently, genes negatively correlated with survival time were selected from the above 19 genes, which together with those 10 high-risk genes retained after univariate analysis and lasso regression, were imported into the CMap to screen the top 25 candidate compounds with the highest scores and display their functions.

### 2.15. Statistical Analysis

The IMvigor210CoreBiologies package used in immunotherapy cohort analysis should be operated in the R 3.6.3 version, whereas all the rest statistical analyses were completed in the R 4.0.5 version (the software packages used included corrplot, limma, GDCRNATools, ggplot2, ggpubr, ggExtra, ggfortify, glmnet, vioplot, pheatmap, rms, reshape2, UpSetR, survival, survminer, preprocessCore, timeROC, clusterProfiler, pRRophetic, maftools, GSEABase and GSVA).

## 3. Results

### 3.1. Differential Expression Analysis

**Figure 2:**
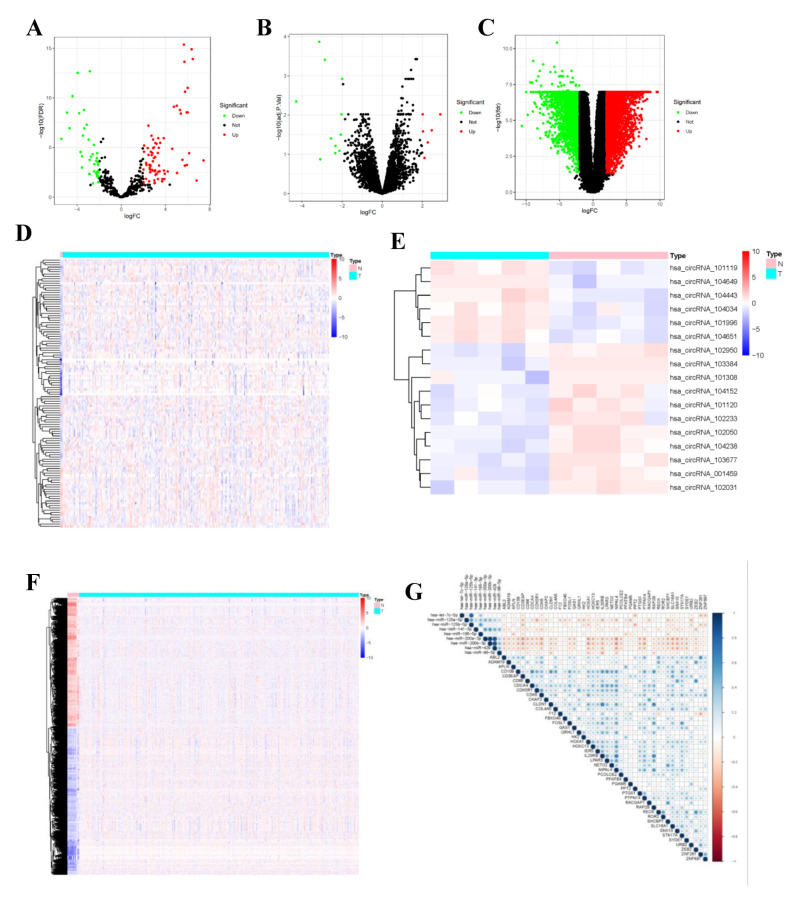
Identified DEcircRNAs, DEmiRNAs, and DEmRNAs. Provided are the volcano plot (**A**) and the heatmap (**B**) of 118 differentially expressed miRNAs between the two groups; the volcano plot (**C**) and the heatmap (**D**) of 7 differentially expressed cirRNAs between the two groups; and the volcano plot (**E**) and the heatmap (**F**) of 6136 differentially expressed protein-coding genes between the two groups; All differentially expressed genes had a log (fold-change) > 2.0 or <−2.0 and the overall of FDR < 0.05. Red represents upregulate and green represent downregulate. The change in color represents the difference in expression. DE, differentially expressed. The heat map showing the correlations of the circRNA-miRNA (**G**), with blue representing positive correlations, red representing negative correlations.

Differential expression analysis was completed by comparing circRNA expression in CC and normal para-carcinoma samples in a GEO cohort. Using the threshold of |log 2(FC)| > 2, altogether 7 DEcircRNAs (6 up-regulated and 11 down-regulated ones) were found to be consistent with GSE102696 dataset. Thereafter, upon the thresholds of FDR < 0.05 and |log 2(FC)| > 2, 118 DEmiRNAs (89 up-regulated and 29 down-regulated ones) (Supplementary Table S2) and 6136 differentially expressed transcriptome genes (3282 up-regulated and 2854 down-regulated ones) (Supplementary Table S1) were identified from the miRNA and mRNA expression data analyzed with “limma” package. In the volcano plot, *FP236383.2* gene (logFC = −11.24, *p* = 5.92 × 10^−37^) exhibited extremely significant difference compared with other genes. This gene was not displayed in the volcano plot to make the volcano plot more beautiful (Figure 2).

### 3.2. Construction of the ceRNA Network and Survival Analysis

**Figure 3:**
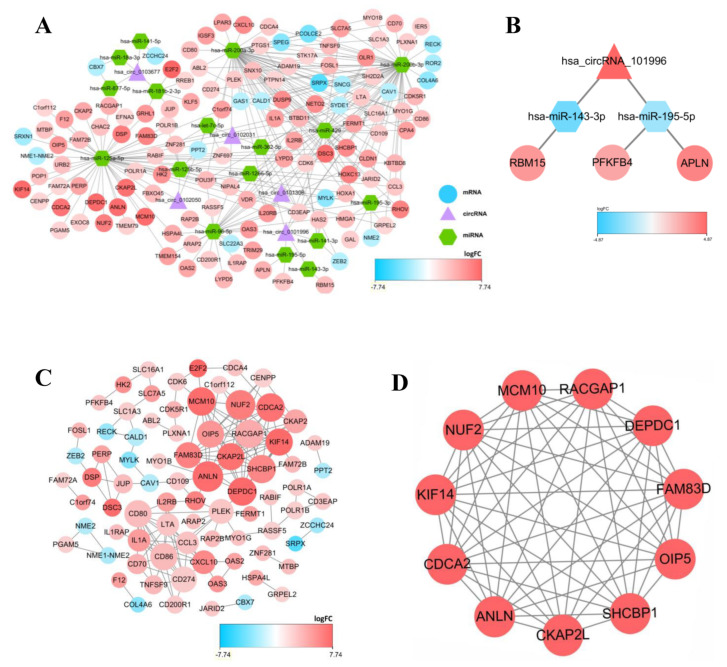
The competing endogenous (ceRNA) network in CC and protein-protein interaction (PPI) network, as well as top 1 modules. (**A**) The circRNA-miRNA-mRNA ceRNA network, in where circRNAs, miRNAs, and mRNAs are represented as triangle, hexagon and circle, respectively. (**B**) The circRNA-miRNA-lncRNA ceRNA network, in where the expression level showed a high-low-high phenomenon. (**C**) protein-protein interaction (PPI) network. (**D**) Top 1 hub module was identified by Cytoscape plug-in MCODE. The size of the dots represents the regulatory capacity of the mRNA, and the larger dots, the stronger the regulatory capacity.

**Figure 4:**
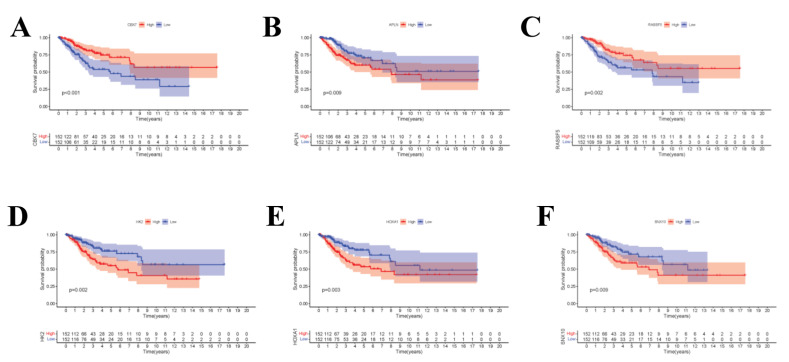
(**A**–**F**) The K–M survival curves of the circRNA-related mRNAs based on the expression, where showed genes significantly correlated with prognosis (*p*-value < 0.01).

To display the relations among DEcircRNAs, DEmiRNAs and DEmRNAs, this work constructed the circRNA-miRNA-mRNA ceRNA network constituted by 151 nodes (including 5 circRNAs, 17 miRNAs and 129 mRNAs) (Figure 3A). In the network diagram, the “up-down-up” expression phenomenon of mRNAs-miRNAs-circRNAs relation axis was found, which was listed separately (Figure 3B). This *circRNA_101996*-based relation axis was not only supported by the experimental results in the relation database, but also displayed corresponding expression trend in CC samples, which was suitable for further experimental validation. Moreover, the K-M survival curve was plotted to evaluate the impact of ceRNA network nodes on the survival rate of CC patients. We discovered that 6 mRNAs (*p*-value < 0.001) were highly correlated with the OS of CC patients (Figure 4).

### 3.3. Construction of the PPI Network

In the PPI network constructed based on the ceRNA network, the top 7 most significant proteins were *MCM10*, *NUF2*, *CDCA2*, *RACGAP1*, *ANLN DEPDC1* and *SHCBP1*, which might have critical roles in CC occurrence and development (Figure 3C,D). In total, 6 clusters were generated in MCODE, and one module with the highest score calculated in MCODE was selected as the hub cluster.

### 3.4. Functional Enrichment Analysis

**Figure 5:**
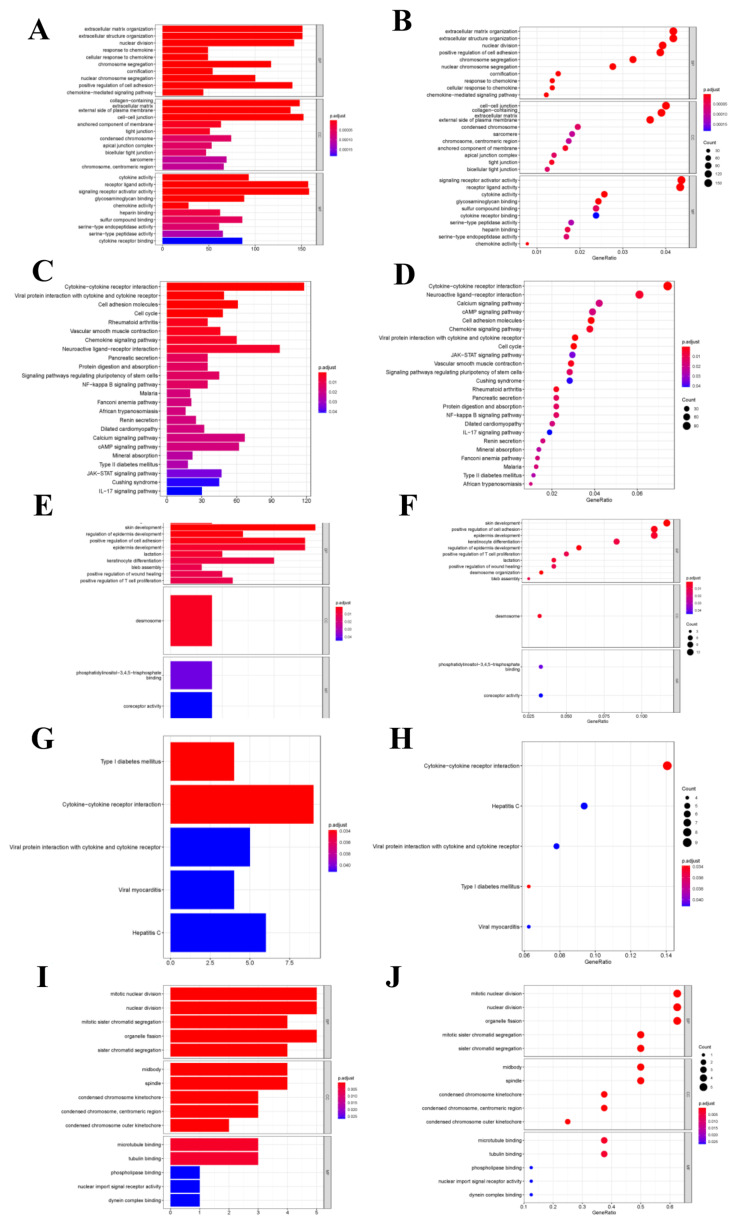
Functional enrichment analysis. (**A**,**B**) GO function enrichment analysis for differential transcriptome genes. (**C**,**D**) KEGG pathway analysis for differential transcriptome genes. (**E**,**F**) GO function enrichment analysis for net-node genes. (**G**,**H**) KEGG pathway analysis for net-node genes. (**I**,**J**) GO function enrichment analysis for hub-cluster genes.

As for differentially expressed transcriptome genes, GO functional annotation results suggested that different expression genes (DEGs) were mainly enriched into “Signal receptor activator activity”, “Extracellular structure and organization” and “Cell-cell connection” functions (Figure 5A–D). KEGG pathway enrichment analysis revealed that node genes were mainly enriched into the “Cytokine-cytokine receptor interaction pathway”, which was closely related to tumor metastasis and development; besides, network node genes were mainly enriched into this pathway (Figure 5E–H). The hub genes were mainly enriched into tumor cell division-related functions, such as mitosis and karyokinesis (Figure 5I,J). But hub genes were not enriched into any KEGG pathway.

### 3.5. Gene Cox Proportional Hazard Regression Model

**Figure 6:**
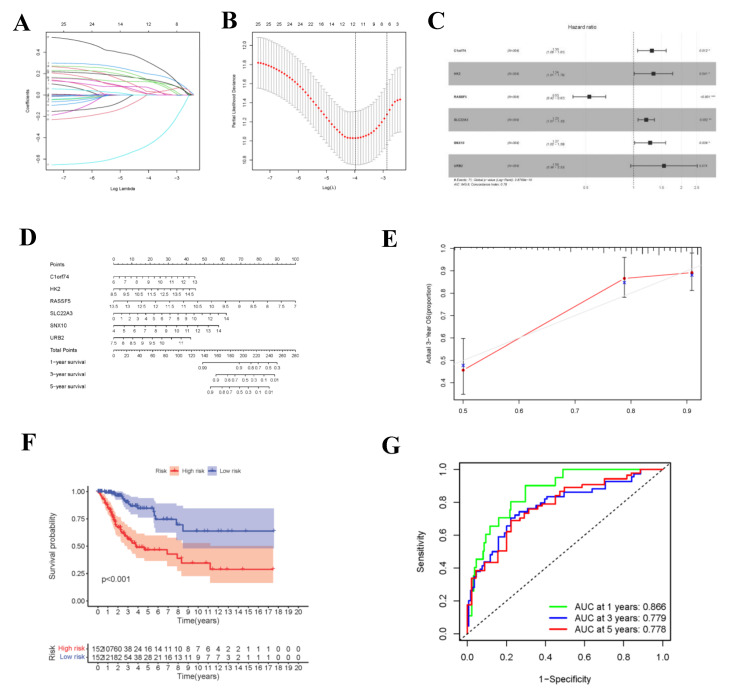
Construction of circ-related prognosis model. (**A**,**B**) The results of lasso regression analysis results. (**C**) Forest plot shows prognostic circ-related mRNA signatures of CC. Six potential prognosis related RNAs were integrated into the Cox proportional hazards regression model (* *p* < 0.05; ** *p* < 0.01; *** *p* < 0.001). The nomogram (**D**) was constructed based on the model. The ROC (**G**) and calibration curves (**E**) indicate the acceptable accuracy (AUCs of 1, 3, and 5-year survivals: 0.866, 0.779, and 0.778) and discrimination of the nomogram. (**F**) OS of patients with high/low-risk score.

**Figure 7:**
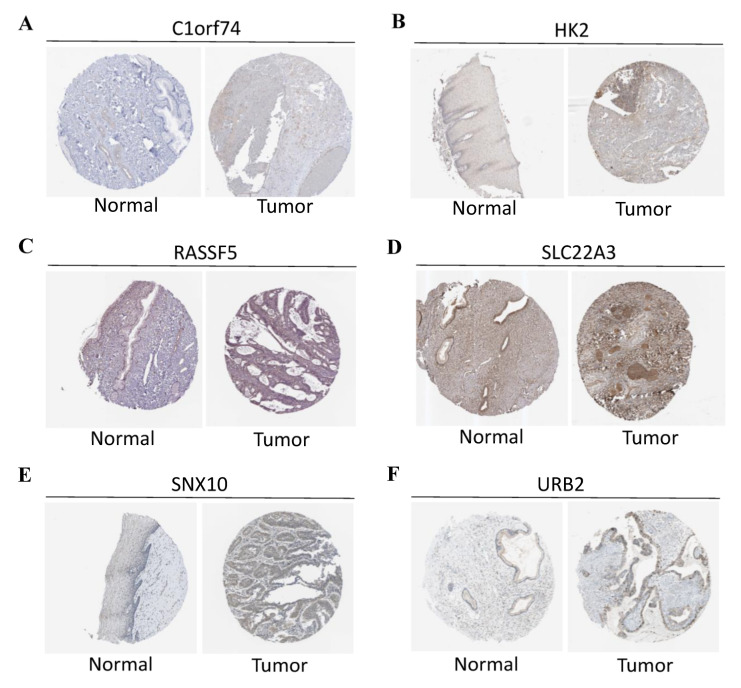
(**A**) Protein levels of C1orf74 in normal cervical tissues (Antibody: HAP028496, Staining: undetected, Quantity: none). Protein levels of C1orf74 in CESC tissues (Antibody: HAP028496, Staining: undetected, Quantity: <25%). (**B**) Protein levels of HK2 in normal cervical tissues (Antibody: HAP028587, Staining: low, Quantity: <25%). Protein levels of HK2 in CESC tissues (Antibody: HAP028587, Staining: low, Quantity: >75%). (**C**) Protein levels of RASSF5 in normal cervical tissues (Antibody: HPA070480, Staining: medium, Quantity: >75%). Protein levels of RASSF5 in CESC tissues (Antibody: HPA070480, Intensity: low, Quantity: <25%). (**D**) Protein levels of SLC22A3 in normal cervical tissues (Antibody: HPA029750, Intensity: medium, Quantity: >75%). Protein levels of SLC22A3 in CESC tissues (Antibody: HPA029750, Intensity: high, Quantity: >75%). (**E**) Protein levels of SNX10 in normal cervical tissues (antibody: HPA015605, Intensity: low, Quantity: 25~75%). Protein levels of SNX10 in CESC tissues (antibody: HPA015605, Intensity: low, Quantity: 75%). (**F**) Protein levels of URB2 in normal cervical tissues (antibody: HPA008902, Intensity: low, Quantity: >75%). Protein levels of URB2 in CESC tissues (antibody: HPA008902, Intensity: medium, Quantity: >75%). (**A**–**F**) The protein expression data covering normal cervical tissues and CESC tissues types was derived from antibody-based protein profiling using IHC.

During the construction of Cox proportional hazard regression model, the model selected after multivariate regression was compared with that after univariate regression alone. After comprehensive consideration, the multivariate regression model was selected. In univariate Cox analysis, 26 genes were selected from the ceRNA network and incorporated into the initial model (Supplementary Figure S1). Lasso regression analysis indicated that 11 genes had the lowest cross-validation points and were of great statistical significance. Subsequently, the Akaike Information Criterion (AIC) value was used to further screen the key genes to construct the 6-gene Cox proportional hazard regression model. Thereafter, a nomogram was established to predict the 1-, 3- and 5-year survival probabilities of CC patients. The area under the ROC curve (AUC) values for 1-, 3- and 5-year survival rates of the model were 0.866, 0.779 and 0.778, respectively, which together with the calibration curve, reflected the nomogram discrimination and accuracy. As revealed by survival curve analysis, the OS of high-risk group was apparently lower than that of low-risk group (*p*-value < 0.001) (Figure 6). This work also searched the experimental data and results against the Human Protein Atlas (HPA) database (https://www.proteinatlas.org, accessed on 12 December 2021), and it was confirmed that the expression levels of *C1orf74*, *HK2*, *RASSF5*, *SLC22A3*, *SNX10* and *URB2* proteins in normal and CC tissues were consistent with our results (Figure 7).

### 3.6. TIIC Levels and Survival Analysis

**Figure 8:**
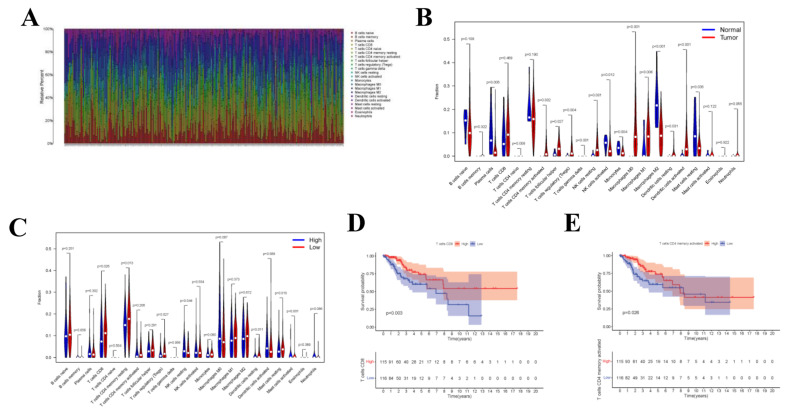
CIBERSORT Estimation and Survival Analysis. The composition (**A**) of immune cells estimated by the CIBERSORT algorithm in 13 normal adjacent tissues and 304 CESC tissues. The violin plot of immune cells (**B**) compares cells’ proportion between the two groups. The violin plot of immune cells (**C**) compares cells’ proportion between the gene high-risk and low-risk groups. (**D**,**E**) The K–M survival curves of immune cells, where showed genes significantly correlated with prognosis (*p* < 0.05).

Upon CIBERSORT analysis, 9 normal tissue samples and 242 tumor tissue samples were selected for subsequent analysis (*p* < 0.05). The proportions of 22 kinds of immune cells in each sample are displayed in the histogram (Figure 8A). According to Wilcoxon rank sum test, Plasma cells (*p* = 0.005), CD4 naive T cells (*p* = 0.008), CD4 memory activated T cells (*p* = 0.002), Regulatory (Tregs) T cells (*p* = 0.004), Gamma delta T cells (*p* < 0.001), Natural killer (NK) cells (*p* = 0.001), Monocytes (*p* = 0.004), Macrophages M0 (*p* < 0.001), Macrophages M1 (*p* = 0.006), Macrophages M2 (*p* < 0.001), and Activated dendritic cells (DCs) (*p* < 0.001) in normal samples were different from those in tumor samples (Figure 8B). To investigate whether the gene risk score affected the immune cell levels, similarly, Figure 8C was drawn to display the differences in immune cell levels between high- and low-risk groups. Results of survival curve analysis indicated that T cells CD8 and T cells CD4 memory activated were related to the OS of CC patients (Figure 8D,E). In addition, TIICs of T stage were also analyzed, and the correlation with clinical stage was also analyzed for the 22 immune cell types (Supplementary Figure S2).

### 3.7. Immune Cell Cox Proportional Hazard Regression Model and Co-Expression Analysis

**Figure 9:**
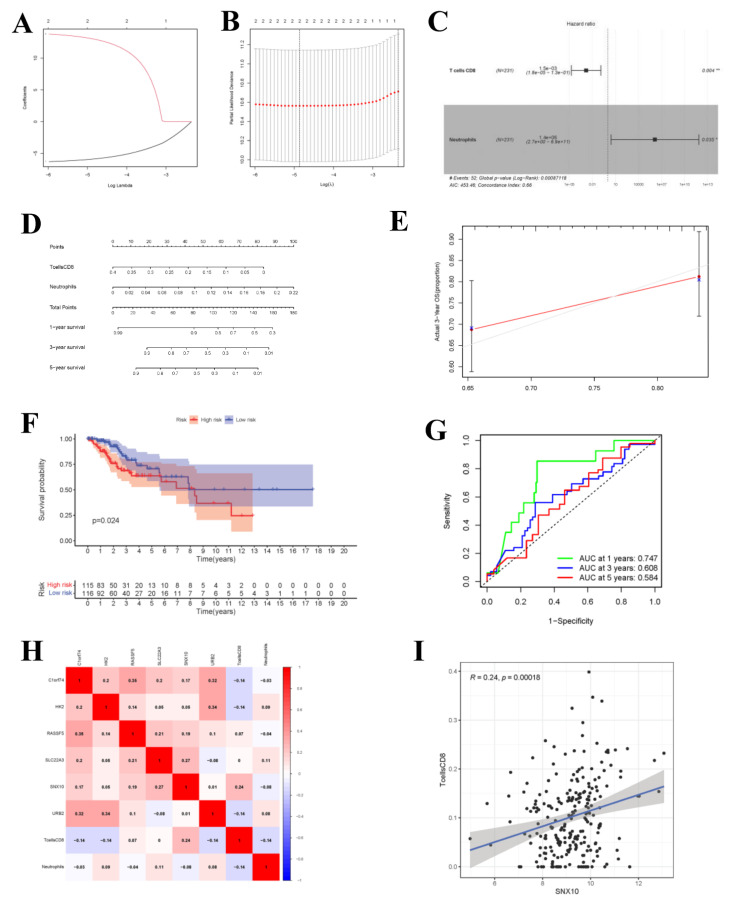
Cox Proportional Hazards Regression Model and Co-expression Analysis. (**A**,**B**) The results of lasso regression analysis results. (**C**) Forest plot shows prognostic circ-related mRNA signatures of CC. T cells CD8 and Neutrophils were integrated into the Cox proportional hazards regression model (* *p* < 0.05; ** *p* < 0.01). The nomogram (**D**) was constructed based on the model. The ROC (**G**) and calibration curves (**E**) indicate the acceptable accuracy (AUCs of 1-year survivals: 0.747) and discrimination of the nomogram. (**F**) OS of patients with high/low-risk score. (**H**) The co-expression heatmap of the RNAs and immune cells in the two Cox proportional hazards regression models; the significant results of Pearson correlation coefficients between the RNAs and immune cells: T cells CD8 and SNX10 (**I**).

Upon univariate Cox regression analysis, 2 immune cell types (CD8+ T cells and Neutrophils) were selected. Afterwards, the model was further optimized by adopting Lasso regression analysis and the AIC value, and CD8+ T cells and Neutrophils were retained for the construction of the final Cox proportional hazard regression model (Figure 9A–F). Then, the 1-, 3- and 5-year OS probabilities of CC patients were predicted by the nomogram plotted based on the model. Both the ROC (AUC value of 1-year survival rate: 0.747) and calibration curves suggested the favorable accuracy of the nomogram. Survival curve analysis demonstrated that, the OS of high-risk group was lower than that of low-risk group (*p*-value < 0.001). The co-expression relations of key genes with immune cells of the two models are displayed in Figure 9H CD8+ T cells were positively correlated with *SNX10* (Figure 9I, R = 0.24, *p*-value < 0.001).

### 3.8. Gene Set Enrichment Analysis

**Figure 10:**
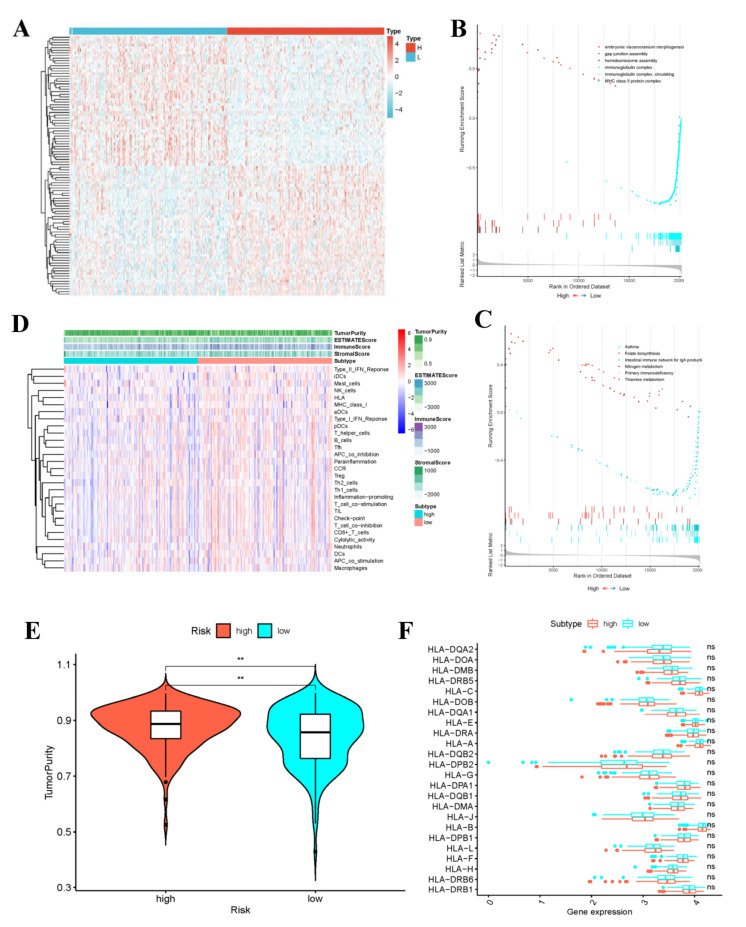
Gene set enrichment analysis and the low-risk and high-risk samples show different tumor purity. (**A**) Differential Analysis Heatmap. (**B**) Plot of GO enrichment results. (**C**) Plot of KEGG enrichment results. The immune status (**D**), tumor purity (**E**) and the distribution of HLA related genes (** *p*-value < 0.01) (**F**) of the low-risk and high-risk samples.

The variation analysis results between the high- and low- risk groups are shown in Figure 10A. The transcript information of high- and low-risk patients classified according to the risk score was analyzed by GSEA. Among the low-risk patients, the representative KEGG pathways (Supplementary Table S3) contained “primary immune deficiency” and “IgA-produced intestinal immune network”. For the high-risk patients, the typical KEGG pathways included “Folic acid biosynthesis”, “Nitrogen metabolism” and “Thiamine metabolism” (Figure 10B). The typical GO functions in low-risk patients were “Immunoglobulin complex”, “Circulation of immunoglobulin complex”, and “MHC II (major histocompatibility complex II)-type protein complex” (Figure 10C). By contrast, the representative GO functions in high-risk patients included “Embryonic visceral skull morphogenesis”, “Hemi-desmosome assembly”, and “Gap junction assembly”. The highly significant pathways and functions in low-risk group were related to immunity, which might provide a certain direction for the immune targeted therapy of CC patients.

### 3.9. Evaluation of Immune Activities of Low- and High-Risk Groups

Through ssGSEA, the expression patterns of 29 immune gene sets in high- and low-risk groups were analyzed, respectively. The heatmap displayed that the ESTIMATE score, ImmuneScore and StromalScore in low-risk group were generally higher than those in high-risk group (Figure 10D). Further, Figure 10E indicated the difference in tumor purity between low- and high-risk groups. To be specific, the low-risk group not only exhibited higher immune activity, but also had apparently decreased tumor purity. Due to the crucial roles of HLA-related genes in immunoregulation, this work conducted further differential analysis. As a result, there was no significant difference in the expression of HLA gene family between high- and low-risk groups (Figure 10F). Subsequently, results of TIDE algorithm analysis indicated that the TIDE scores between high- and low-risk groups were not significantly different, but the low-risk group was associated with higher probabilities of CD8 expression and T cell dysfunction. The boxplot results were corroborated by the scatter diagram mutually (Supplementary Figure S3).

### 3.10. Mutation Analysis

**Figure 11:**
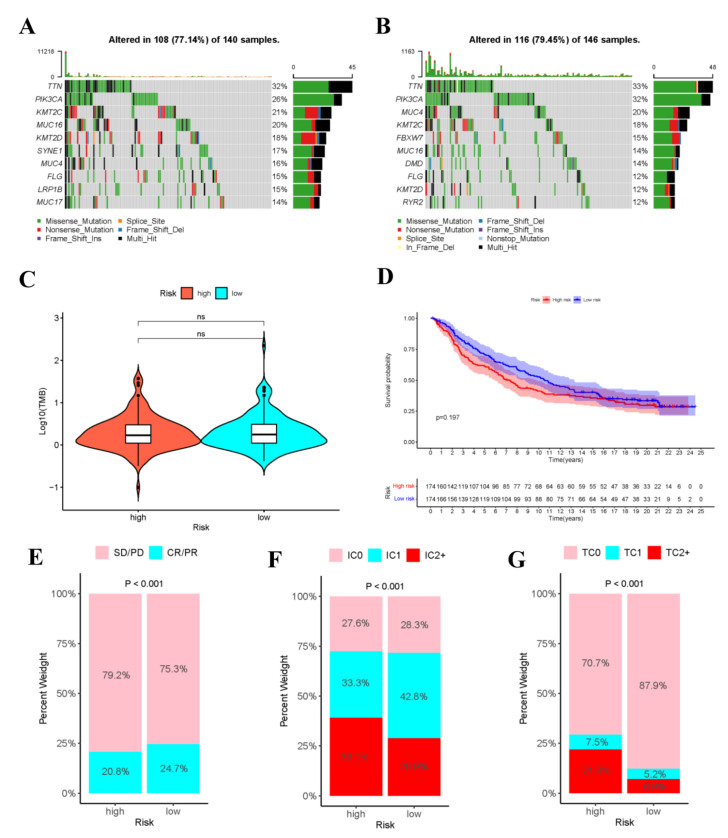
Analysis of tumor mutations and immunotherapy cohort analysis. Oncoplot shows the sample gene mutation landscape for the high-risk (**A**) and low-risk (**B**) groups, the genes are sorted according to their mutation frequency. Violin plots shows no difference in TMB between high- and low- risk groups (**C**). (**D**)The K–M survival curves of high- and low- risk groups in the IMvigor210 Cohort. (**E**) Rate of clinical response (complete response [CR]/partial response [PR] and stable disease [SD]/progressive disease [PD]) to anti-PD-L1 immunotherapy in high- or low-risk groups in the IMvigor210 cohort. (**F**) Level of IHC-assessed PD-L1 staining on immune cells (IC) in high- or low- risk groups in the IMvigor210 cohort, with IC0 meaning < 1 percent, IC1 ≥ 1 percent but <5 percent, IC2+ ≥ 5 percent immune cells staining for PD-L1. (**G**) Level of IHC-assessed PD-L1 staining on tumor cells (TC) in high- or low-risk groups in the IMvigor210 cohort, with TC0 meaning <1 percent, TC1 ≥ 1 percent but <5 percent, TC2+ ≥ 5 percent tumor cells staining for PD-L1.

Gene mutation is an important cause of the production of cancer cells. Therefore, this work analyzed the risk score-stratified TMB of high- and low-risk groups (Figure 11A,B). The order of cell mutations in high-risk group was as follows, *TTN* (32%) > *PIK3CA* (26%) > *KMT2C* (21%) > *MUC16* (20%) > *KMT2D* (18%) > *SYNE1* (17%) > *MUC4* (16%) > *FLG* (15%) > *LRP1B* (15%) > *MUC17* (14%) (Figure 11), while that in low-risk group was *TTN* (33%) > *PIK3CA* (32%) > *MUC4* (20%) > *KMT2C* (18%) > *FBXW7* (15%) > *MUC16* (14%) > *DMD* (14%) > *FLG* (12%) > *KMT2D* (12%) > *RYR2* (12%) (Figure 11). Noteworthily, the commonly seen *TP53* mutation was seen among the top 10 mutations in high- or low-risk populations. In addition, Figure 11C revealed no significant difference in TMB between high- and low-risk populations.

### 3.11. Immunotherapy Cohort Analysis

To explore the role of risk score model in predicting the therapeutic benefits of patients, immunotherapy cohort analysis was conducted. As shown in the figure, in the IMvigor210 cohort, the tumor cell-positive score (TPS) and immune cell-positive score (IPS) in high-risk group significantly increased compared with those in low-risk group, and the objective response rate (ORR) of anti-PD-L1 immunotherapy was lower than that in low-risk group. These data indicated that risk score was probably related to the response to immunotherapy (Figure 11E–G). Additionally, the high- and low-risk groups in IMvigor210 cohort were performed survival analysis (Supplementary Table S4), as shown in the Figure 11D.

### 3.12. Drug Sensitivity Analysis

Apart from immunotherapy, this work also analyzed the significant difference in the sensitivity to the same drug between high- and low-risk groups. Altogether 24 more effective drugs for high-risk patients were chosen, including Trametinib (*p* = 4.1 × 10^−8^), YM155 (*p* = 1.8 × 10^−7^), TAE684 (*p* = 4.4 × 10^−6^), FTI-277 (*p* = 6 × 10^−6^), Docetaxel (*p* = 1 × 10^−5^), XAV939 (*p* = 2 × 10^−5^), Elesclomol (*p* = 2.9 × 10^−5^), JNK-9L (*p* = 3.8 × 10^−5^), Thapsigargin (*p* = 3.9 × 10^−5^), Cetuximab (*p* = 4.7 × 10^−5^), PD-0325901 (*p* = 6.9 × 10^−5^), and Midostaurin (*p* = 7.3 × 10^−5^) (Supplementary Figure S5).

### 3.13. Candidate Compounds in Cmap

**Figure 12:**
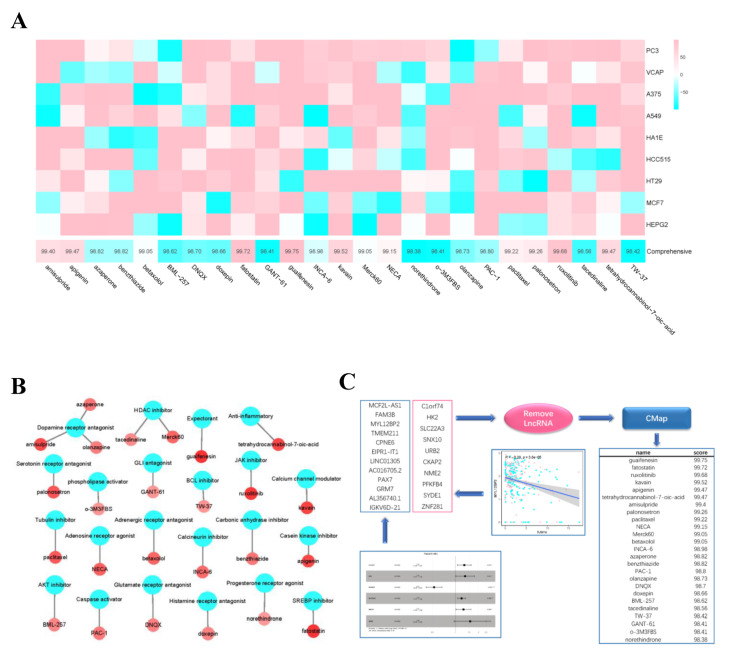
Drug prediction. (**A**) the abscissa of the heatmap is the compound and score, the ordinate is the cell line, red indicates sensitivity to the compound, and blue indicates insensitivity to the compound. (**B**) Network diagram shows compounds and their functional classification. (**C**) The gene set consists of two parts, one is the genes filtered by univariate Cox analysis and lasso regression analysis, and the other is the genes that are significantly different between high- and low- risk groups and negatively correlated with survival time. CMap analysis are performed after removal of non-coding RNA, then obtain the top 25 drugs (* *p* < 0.05; ** *p* < 0.01; *** *p* < 0.001).

We identified 19 genes that were both differentially expressed in the high- and low- risk groups and correlated with survival time. Then we combined these genes with the single-factor filter genes and fed them into Cmap. The top 25 candidate compounds in the result (Figure 12C) included guaifenesin (Comprehensive = 99.75), fatostatin (Comprehensive = 99.72), ruxolitinib (Comprehensive = 99.68), kavain (Comprehensive = 99.52), apigenin (Comprehensive = 99.47), and tetrahydrocannabinol-7-oic-acid (Comprehensive = 99.47). The screened compounds were mainly the receptor inhibitors or antagonists. Compounds with higher scores showed better reliability (Figure 12A,B).

## 4. Discussion

In recent years, the morbidity of CC shows a decreasing trend on the whole, but its poor prognosis remains the most common problem [23]. With the development of cell molecular biology and immunology, immunotherapy has become a new treatment option, which has opened a new door for tumor treatment. Some existing studies indicate that ceRNAs play critical roles in the molecular regulation of tumor genesis and development and the difference in TIIC levels in multiple tumors [24]. circRNAs have been verified to participate in the physiological and pathological processes of tumor genesis and development, which affect the resistance to immunotherapy, targeted therapy and chemotherapy [25,26,27]. So far, microarray and high-throughput sequencing techniques have been utilized to identify the potential therapeutic targets of CC. Previous studies usually construct single populations or have small sample sizes, which has restricted their reliability [28]. This study integrated samples from 3 databases to investigate the roles and ceRNA network and immune infiltration in CC as well as the potential regulatory mechanism, and to explore the key prognostic biomarkers. Due to the emergence of immunotherapy, extensive immune and drug sensitivity analyses were conducted in the high- and low-risk populations, and the potential drugs were screened. In addition, we also carried out mutation analysis and immunotherapy cohort analysis to explore the impact of the risk score model on the response to immunotherapy.

In our constructed initial ceRNA network, 5 circRNAs (*hsa_circ_0101996*, *hsa_circ_0101308*, *hsa_circ_0102050*, *hsa_circ_0102031*, and *hsa_circ_0103677*) were selected as the “sponges” to adsorb 17 miRNAs, thus indirectly regulating the expression levels of 129 mRNAs through isolating target miRNAs. To obtain the more important circRNAs, the circRNA-miRNA-mRNA relation axis showing the “up-down-up” expression phenomenon was screened from the network diagram to establish a novel network, of which, *Hsa_circ_0101996* exhibited an important role. *Hsa_circ_0101996* is originated from SOS2 (SOS Ras/Rho guanine nucleotide exchange factor 2), extensively expressed in diverse tissues and mediates multiple signaling pathways that promote Ras activation [29,30]. The Ras-mediated signaling pathway regulates multiple biological functions, including carcinogenesis [31]. Therefore, the regulatory axis shown in Figure 3B is of high value for further study. In addition, the functional enrichment of node genes revealed that the functions of the entire network were mainly focused on cell recognition, cell adhesion, and epidermal growth meaning that our constructed network seems to hide a large number of regulatory axes of cancer cell spread which are worth further exploration. Based on the ceRNA network, this work also constructed the PPI network and selected some genes with high connectivity. From the results of functional enrichment, these hub genes are closely related to fission and organelle division which are consistent with their high expression in CC patient species and also further indicates that our constructed ceRNA plays an important role in tumorigenesis. In particular, we found that these central genes were centrally regulated by has-miR-125a-5p which has high prognostic value in CC species was reported last year [32]. Our study more broadly and deeply explains the important role of it in CC. Moreover, *FAM83D*, *DEPDC1*, *CDCA2*, *CKAP2L* and *SHCBP1* have been reported to participate in the genesis and development of other tumors. The hub genes discovered in these PPI networks may have important functions in CC, which should be further investigated. Numerous studies have indicated the differential expression of circRNAs in CC, which is related to the clinicopathological features of CC patients [33].

In this study, 2 prognostic models were constructed, and 2 prediction nomograms were plotted, which consisted of 6 genes (*C1orf74*, *HK2*, *RASSF5*, *SLC22A3*, *SNX10* and *URB2*) and 2 immune cell types (CD8+ T cells and Neutrophils), respectively. The results suggested that, the high expression of *C1orf74*, *HK2*, *SLC22A3*, *SNX10* and *URB2* and Neutrophils, as well as the low expression of *RASSF5* and CD8+ T cells was related to the poor prognosis of CC patients, which might serve as the key prognostic biomarkers of tumor. The key factors in the 2 nomograms were subject to correlation analysis, which indicated that CD8+ T cells were positively correlated with *SNX10*. Therefore, it was speculated that *SNX10* and CD8+ T cells might have important roles in the genesis and treatment of CC. After literature review on these 6 genes, it was found that only *C1orf74* was not reported to be related to cancer. Furthermore, as suggested by co-expression analysis results, *C1orf74* was significantly positively correlated with other genes in the model, like *RASSF5* (cor > 0.35) and *URB2* (cor > 0.32). Therefore, the role of *C1orf74* in CC occurrence deserves further investigation. Interestingly, *HK2*, *SLC22A3* and *SNX10* have been reported to be related to CC. Jiang P et al. discovered that *SNX10* was an independent prognostic factor for OS of CC patients, which was highly expressed in tumor tissues [28]. Wang Q et al. reported that METTL3 enhanced the *HK2* stability through the YTHDF1-mediated m6A modification, thus promoting the Warburg effect of CC, which might shed new light on CC treatment [34]. Additionally, Xiao H et al. indicated that *SLC22A3* up-regulation predicted the dismal prognosis of CC patients [35]. However, although the overexpression of *SLC22A3* is related to the high mortality risk of CC patients, its expression levels in normal cervical cells are dramatically higher than those in cancer patients. This means that the mechanism by which *SLC22A3* affects CC is relatively complex, and more research is warranted. *RASSF5* is referred to as a tumor suppressor factor, and it is the pro-apoptotic component of Ras, which can induce the p53-mediated cell apoptosis [36,37]. Some reports have discovered the *RASSF5* inactivation phenomenon in multiple human cancers, including lung cancer and gastric cancer. Similar to the above reports, this study illustrated that *RASSF5* down-regulation was related to the poor prognosis of CC patients. In our constructed nomograms, the line representing the risk score of *RASSF5* was the longest, far longer than those of the other 5 genes. It implied that even a slight decrease in *RASSF5* expression substantially affected the patient survival and greatly increased the risk. Our research further proved that *RASSF5* acted as a tumor suppressor gene.

The immune cell levels in samples were analyzed by using the CIBERSORT algorithm. As a result, there were significant differences in cell levels between normal and tumor samples, like γδ T cells, Macrophages M0, Macrophages M2, and Activated DCs. Typically, γδ T cells, Macrophages M0, and Activated DCs were more abundant in tumor samples, whereas Macrophages M2 had a higher level in normal samples. γδ T cells can infiltrate into diverse tumor tissues, exert the cytotoxicity to kill tumor cells, participate in immunoregulation, and present the antigen. In addition, they can recognize different tumor cells in the major histocompatibility complex (MHC) non-restricted manner, and promote cancer cell division through producing chemokines and cytokines or through the direct contact with cancer cells via the death receptor signal [38,39]. This suggests that γδ T cells may have potential effects on antitumor immunotherapy. Consequently, the role of γδ T cells in anti-CC immunotherapy should be further explored. In previous studies, Macrophages M0 have been demonstrated to be correlated with CC prognosis [40,41]. DCs are a special type of antigen presenting cells (APCs), which have pivotal roles in the congenital and adaptive immune responses. Besides, they are tightly related to tumor immunology and immunotherapy, and they have been verified to be closely associated with CC treatment. This study screened CD8+ T cells and neutrophils to construct the Cox proportional hazard regression model. The ROC results suggested definite clinical value. Similar to our research, some studies discover that high CD8+ T cell infiltration level is significantly related to the favorable prognosis of patients with cervical squamous cell carcinoma and endocervical adenocarcinoma (CESC), and neutrophil infiltration level is negatively correlated with long-term survival [42,43]. CD8+ T cells are the major component in the TIICs. Consequently, regulating CD8+ T cell response has always been the focus of cancer immunotherapy [44]. Neutrophils account for about 60% of all white blood cells, and they are considered as the first line of defence against inflammation and infection [45]. Neutrophil infiltration is found in multiple tumor tissues, and tumor-associated neutrophils (TANs) are related to the advanced disease in cancer patients. The aforementioned results support our analysis.

As observed from GSEA results, the differences in functional enrichment between high- and low-risk groups were mainly manifested in immunity and cell connection. Typically, the enrichment of functions like immunoglobulin assembly and MHC was more apparent than that in low-risk group. ssGSEA results further verified the difference in immunity level between high- and low-risk groups, and high-risk patients had lower immune scores and higher tumor purity. CD8+ T cells played a pivotal role in CC immunity. TIICs, GSEA, ssGSEA and TIDE algorithm results repeatedly validated that TIIC levels were negatively correlated with risk score, which will provide assistance in the treatment and prediction of CC. Different from CD8+ T cells, there was no significant difference in HLA gene family between high- and low-risk groups. To sum up, we can confirm that the content of CD8+ T cells is an excellent prognostic marker of CC.

To explore the significance of risk score model in the immunotherapeutic effect, this work analyzed the TMB of high- and low-risk groups. Some previous studies have conducted mutation analysis on somatic cells in CC tumor samples [46]. Therefore, this work classified samples into high- and low-risk groups on this basis. Six of the top 10 mutant genes obtained were consistent with that research. We discovered that, research results from multiple CC mutation analysis studies include *TTN*, *PIK3CA*, *MUC4*, *MUC16* and *FBXW7* genes. Typically, *TTN* was the most common mutant gene in this study. *TTN* mutation can serve as the predicting factor for ICB immunotherapy to predict the long-term survival of pan-solid tumors like CC, and provide clinically feasible evaluation on TMB and ICB treatment outcome [47]. *PIK3CA* mutation results in the increased proliferation of CC cells and the decreased cell apoptosis. In HeLa cells, knockdown of *MUC-4* significantly reduces the invasiveness and mesenchymal characteristics of CC cells [48]. Multiple RNAs can target *FBXW7* to affect the proliferation, migration and invasion processes of CC cells, which are suppressed by *MUC16* knockdown [49]. The known functions of these immune-related genes in cancer suggest that the mutations of these genes play crucial roles in the immunoregulation of CC, which deserves further verification. Apart from the above-mentioned reported common mutant genes, the potential roles of infrequent CC mutation-related genes like *KMT2C*, *DMD*, *FLG*, *KMT2D* and *RYR2* should also be explored.

In recent years, the PD-L1 ICI immunotherapy has received wide attention, and it has become the ground-breaking innovative treatment for malignant tumor. It has been suggested that patients with higher PD-L1 expression gain more benefits from immunotherapy, and PD-L1 expression has become a favorable biomarker for guiding the selection of treatment scheme in clinic. In our study, the proportions of PD-L1 membrane-stained tumor cells and immune cells significantly increased in high-risk group, and the Objective Response Rate (ORR) in high-risk group was low upon solid tumor therapeutic effect evaluation analysis. So to some extent the high-risk group is more suitable for this immunotherapy [50]. In addition, a study indicates that PD-L1 promotes apoptosis of CD8+ T cells. In our study, the level of CD8+ T cells in CC patients from the high-risk group was significantly lower than that in the low-risk group, whereas PD-L1 expression predicted by IMvigor210 were opposite, which are consistent with previous study in gastrointestinal stromal tumours. However, because the IMvigor210 were from urothelial carcinoma, further validation experiments are needed.

We selected the CC immunotherapy-related drugs from the known drugs in chemotherapy and targeted therapy. Compared with low-risk group, there were 24 drugs in high-risk groups with low IC50 values. Of them, Trametinib and TAE684 have been reported to be used in immunotherapy or targeted therapy of multiple cancers. YM155 can be used in combination with tumor necrosis factor-related apoptosis-inducing ligand (TRAIL) to induce sensitivity of HeLa cells at a low dose, thus effectively treating CC [51]. FTI-277 can suppress tumor cell invasion and migration through multiple mechanisms. Docetaxel can be used in combination with multiple chemotherapeutic agents for the immunotherapy of CC, among which, the Sal-Docetaxel-loaded gelatinase-stimulated nanoparticles (Sal-Doc NPs) can significantly suppress CC cell growth [52]. The risk score model in this study might be the potential predicting factor for the sensitivity of chemotherapeutic and targeted therapeutic drugs, and the screened drugs might provide a new scheme for the treatment of CC patients. Moreover, we also predicted the potential compounds or drugs that might treat CC. Sterol regulatory element binding protein-2 (SREBP-2) and its regulatory enzyme participate in the progression of diverse cancers and act as the potential targets. Therefore, as an SREBP inhibitor, fatostatin has been reported to be used in combination with other drugs for the treatment of different cancers [53]. Ruxolitinib is the inhibitor of Janus kinase 2 (JAK2), which can synergistically induce cell apoptosis with cisplatin, and is promising to provide a method to treat CC patients [54]. In the epithelial tumor test (ETT) on black fruit flies, kavain bound to the chemotherapeutic doxorubicin (DXR) to synergistically induce the enhanced carcinogenic effect of DXR on tumor [55]. These predicted drugs have high reliability and are related to cancer treatment. In future studies, the roles and mechanisms of action of these drugs in the treatment of CC patients can be further explored.

This study integrated data from 3 databases, which decreased the experimental error induced by small sample size to some extent. When screening the network nodes, the nodes were conducted correlation analysis, which improved the experiment accuracy. Besides, extensive immunological analyses were performed to explore the relation of the risk score model with immunotherapy. This work also screened sensitive drugs from the known drugs for high-risk CC patients, while predicting the potential immunotherapeutic agents for CC patients. Certainly, certain inevitable limitations should be noted in this work. Firstly, our research data were derived from the public databases, and some of the data parameters might be incomplete, leading to the potential risks of error and deviation. Secondly, the circRNA sample size and the miRNA normal sample size were small, and there might be occasional results in their differential analysis. Finally, in this study, the construction of regulatory networks and models and the prediction of therapeutic agents were dependent on a series of bioinformatics algorithms and databases. Therefore, lots of experiments are warranted to verify the accuracy of these prediction conclusions.

## 5. Conclusions

Based on TIICs and ceRNA network, this work constructed two nomograms to predict the overall survival of cervical cancer (CC) patients. From the network, we identifiedhub genes affecting cell proliferation which regulated by has-miR-125a-5p. In the nomograms, the impacts of prognostic model-related genes and cells on CC were elaborated. Thereafter, we completed the GSEA, ICP detection and TIDE algorithm analysis on high- and low-risk groups and confirmed the important role of CD8+ T cells in the prognosis of CC. Moreover, mutation analysis and immunotherapy cohort analysis were completed, which indicated that the risk model was able to predict the response to immunotherapy. In the last part of our study, we screened 24 anti-cancer drugs that high-risk patients were sensitive to and potential compounds that may have targeted therapeutic effects on CC. This study provided related information contributing to the diagnosis and treatment of CC patients, and selected several drugs for reference.

## Data Availability

Publicly available datasets were analyzed in this study. This data can be found here: https://xenabrowser.net/datapages/?cohort=TCGA%20Cervical%20Cancer%20(CESC)&removeHub=https%3A%2F%2Fxena.treehouse.gi.ucsc.edu%3A443 (accessed on 3 October 2021). The data presented in this study are available in Supplementary Tables. The datasets used or analyzed during the current study are available from the corresponding author on request.

## Supplementary Materials

The following supporting information can be downloaded at: https://www.mdpi.com/article/10.3390/curroncol29110633/s1, Supplementary Table S1: Variation analysis results of mRNAs. Supplementary Table S2: Variation analysis results of miRNAs. Supplementary Table S3: Results of GSEA in KEGG. Supplementary Table S4: Results of GSEA in GO. Supplementary Table S5: Results of variation analysis between high and low risk groups. Supplementary Table S6: The RiskScore and high- and low-group assignment of samples from IMvigor210 cohort. Supplementary Table S7: Predictive results of half effective inhibition concentrations of cancer drugs in CC patients. Supplementary Figure S1: The results of univariate Cox analysis and lasso regression analysis. Supplementary Figure S2: Correlation Plot for Immune Cell Clinical Stage. Supplementary Figure S3: TIDE analysis for predicting the likelihood of clinical response to immune checkpoint inhibitors. Supplementary Figure S4: Box plots shows sensitivity of CC patients to known chemo- or immunotherapeutic agents. Supplementary Figure S5: Scatter plots show the correlation between genes with significant differences in high- and low-groups and survival time. Blue for low-risk group and red for high-risk group.
