# Supplementary material for: CircRNA-Based Cervical Cancer Prognosis Model, Immunological Validation and Drug Prediction
Source: Curr Oncol. 2022 Oct 25;29(11):7994–8018. doi: 10.3390/curroncol29110633 (PMC9689098; doi:10.3390/curroncol29110633)
Supplement: Supplementary file 1 [file curroncol-29-00633-s001.zip › Supplementary Figure S2.pptx]

## Slide 1
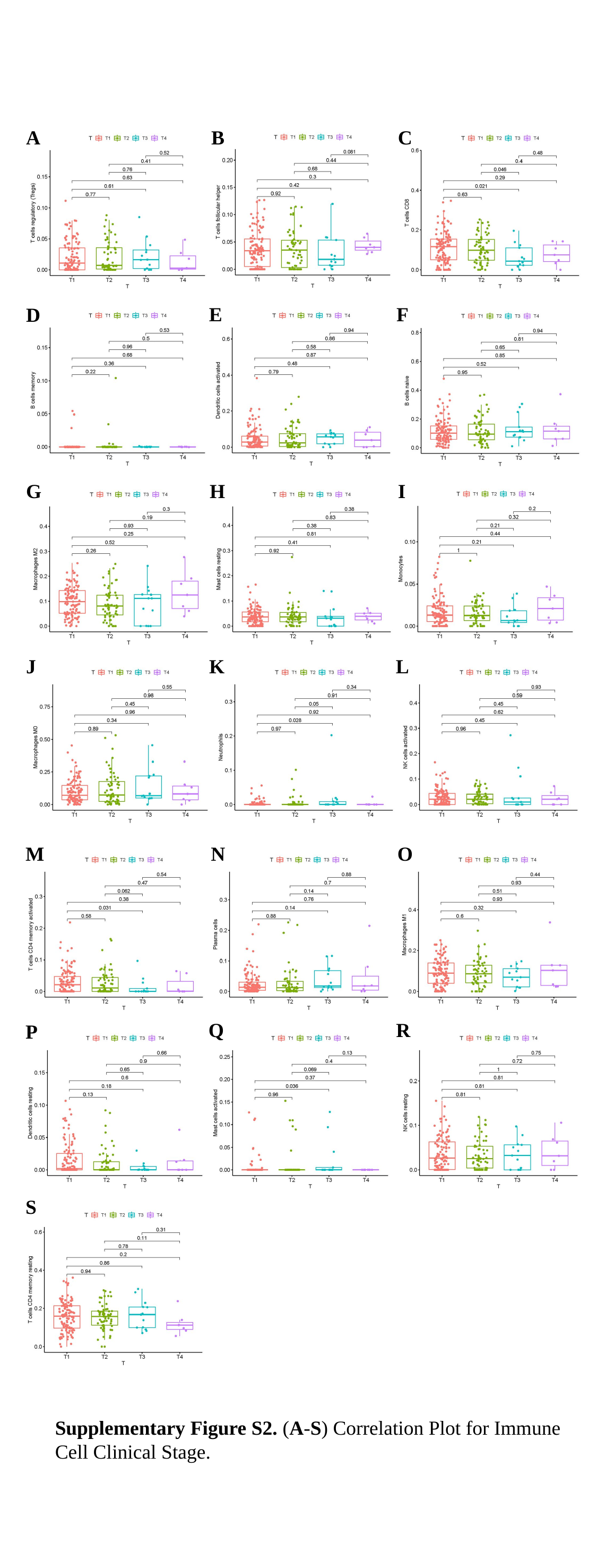

A
C
B
E
F
D
G
H
I
J
K
L
M
N
O
Q
R
P
S
Supplementary Figure S2. (A-S) Correlation Plot for Immune Cell Clinical Stage.
