# Supplementary material for: CircRNA-Based Cervical Cancer Prognosis Model, Immunological Validation and Drug Prediction
Source: Curr Oncol. 2022 Oct 25;29(11):7994–8018. doi: 10.3390/curroncol29110633 (PMC9689098; doi:10.3390/curroncol29110633)
Supplement: Supplementary file 1 [file curroncol-29-00633-s001.zip › Supplementary Figure S5.pptx]

## Slide 1
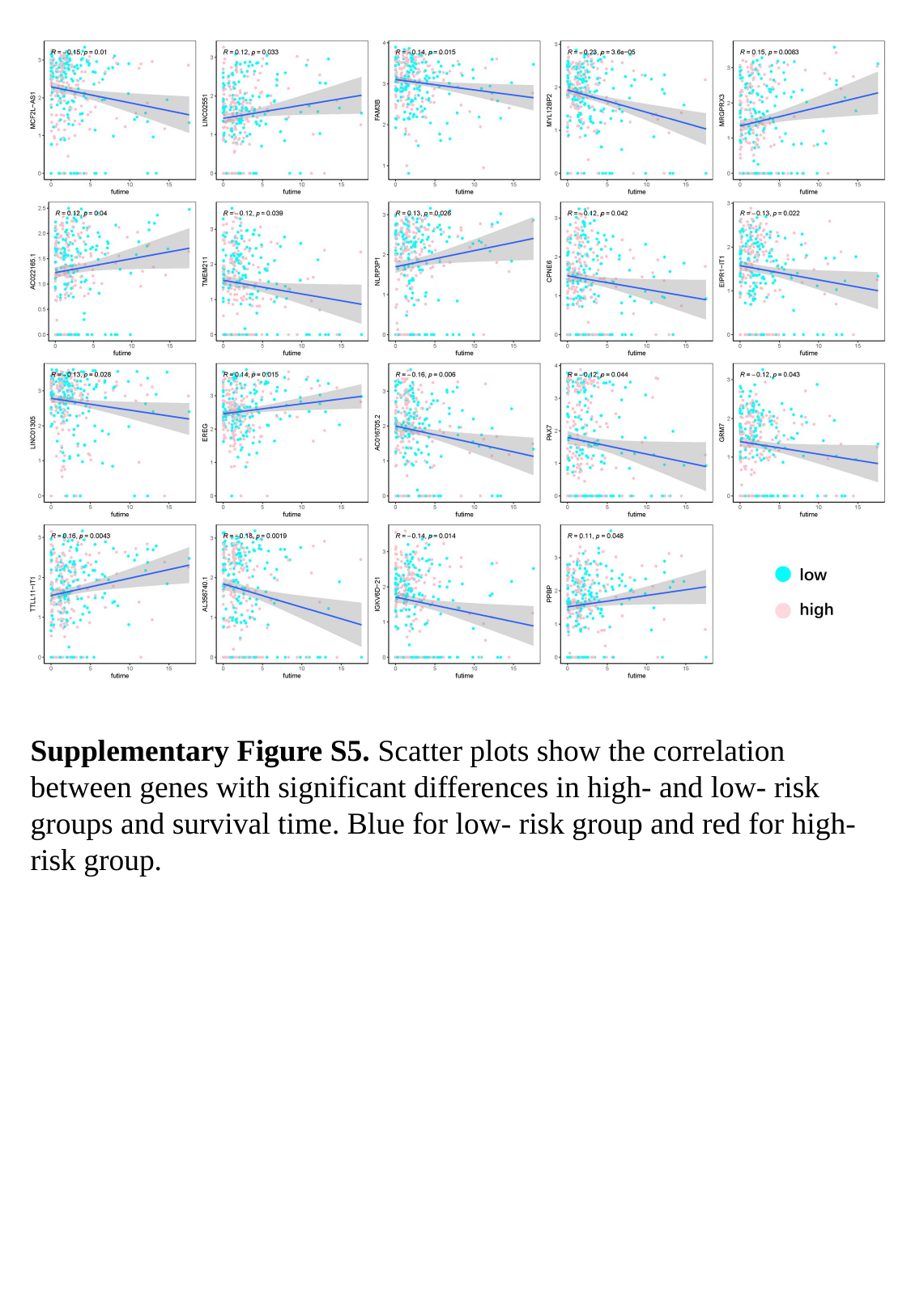

Supplementary Figure S5. Scatter plots show the correlation between genes with significant differences in high- and low- risk groups and survival time. Blue for low- risk group and red for high- risk group.
